# Replicating $\textsc {Restart}$ with Prolonged Retrials: An Experimental Report

**DOI:** 10.1007/978-3-030-72013-1_21

**Published:** 2021-02-26

**Authors:** Carlos E. Budde, Arnd Hartmanns

**Affiliations:** 8 Eindhoven University of Technology, Eindhoven, The Netherlands; 9 Aalborg University, Aalborg East, Denmark; University of Twente, Enschede, The Netherlands

## Abstract

Statistical model checking uses Monte Carlo simulation to analyse stochastic formal models. It avoids state space explosion, but requires rare event simulation techniques to efficiently estimate very low probabilities. One such technique is $\textsc {Restart}$. Villén-Altamirano recently showed—by way of a theoretical study and ad-hoc implementation—that a generalisation of $\textsc {Restart}$ to *prolonged retrials* offers improved performance. In this paper, we demonstrate our independent replication of the original experimental results. We implemented $\textsc {Restart}$ with prolonged retrials in the and modes tools, and apply them to the models used originally. To do so, we had to resolve ambiguities in the original work, and refine our setup multiple times. We ultimately confirm the previous results, but our experience also highlights the need for precise documentation of experiments to enable replicability in computer science.

## 1 Introduction

In stochastic timed systems, the time between faults, customer interarrival times, transmission delays, or exponential backoff wait times follow (continuous) probability distributions. Probabilistic model checking [3] can compute dependability metrics like reliability and availability in the Markovian case. To evade state space explosion and evaluate non-Markovian systems, statistical model checking (SMC [2]) has become a popular alternative. At its core, SMC is Monte Carlo simulation for formal models. It faces a runtime explosion when estimating the probability $p$ of a *rare event* with a sufficiently low error, e.g. an error of $\pm 10^{-10}$ for $p \approx 10^{-9}$ (i.e. a *relative* error of 0.1). *Rare event simulation* (RES) techniques [17] address this problem. They can broadly be categorised into *importance sampling* and *importance splitting*. The former changes the probability distributions while the latter changes the simulation algorithm to make the rare event more likely. Both techniques then compensate for these changes in the statistical evaluation. RES has garnered the interest of mathematicians and computer scientists alike. The scientific outcomes range from theoretical studies of a RES technique's limit behaviour and optimality [8,14,16] over experimental validation on Matlab studies or ad-hoc implementations [10,11,19] to application

* Authors are listed alphabetically. This work was supported by NWO via project no. 15474 (SEQUOIA) and VENI grant no. 639.021.754.

reports using larger case studies [5,12,18] as well as automated tools [4,6,15,18] that accept a loss of optimality in exchange for practicality.

Two recent papers showed theoretically [21] and empirically [19] that *prolonging retrials* in the RESTART importance splitting technique [22] reduces the required number of samples for the same error, with optimal runtime around prolonging by 1 to 2 levels. The models and parameters used in [19] are described in supplementary material [20], but the implementation is not publicly available. In this paper, we demonstrate our *replication* of the results of [19,21], where replication "means that an independent group can obtain the same result using artifacts which they develop completely independently" in the ACM terminology [1]. To this end, we implemented RESTART with prolonged retrials (RESTART-P) in the FIG rare event simulator [4] and the modes statistical model checker [7] of the MODEST TOOLSET [13]. We recreated the models in the IOSA and MODEST languages, and ran experiments following the original setup.

Our experiments confirm the behaviour and performance improvements of RESTART-P reported in [19,21]. However, we encountered ambiguities in the textual and pictorial descriptions of RESTART-P and the experimental setup in the original papers, some of which we could only resolve with input from the author of [19,21]. Different parts of our work thus reside on different levels between replication and *reproduction* (which "means that an independent group can obtain the same result using the author's own artifacts" [1]). Throughout the paper, we document where we achieved fully independent replication, where information from private communication was needed, and where we had to ultimately resort to requesting and inspecting the source code for the original implementation.

The contribution of this paper is thus threefold: (1) We provide pseudocode for RESTART-P in Sect. 2 that clarifies the technical details w.r.t. [19,21]. (2) We demonstrate the new RESTART-P capabilities of FIG and modes by replicating the original experiments in Sect. 3. (3) We reflect on our experience (as practical computer scientists) in independently replicating existing (theoretically-flavoured) work.

## 2 RESTART with Prolonged Retrials

Let a stochastic timed discrete-event model be given as a tuple $\langle S, s_0, \mathit{step}, F\rangle$ of a set of states $S$, an initial state $s_0 \in S$, a function $\mathit{step}\colon S \to [0,\infty) \times S$ where $\mathit{step}(s)$ samples a random path from $s$ to the next event and returns a pair $\langle t, s'\rangle$ of its duration and next state, and a subset of rare event states $F \subseteq S$. A simulation *run* is a sequence of states obtained by repeatedly applying *step*. Models with general probability distributions encode their memory in the states.

Importance splitting uses an *importance function* $f_I\colon S \to [0,\infty)$ indicating "how close" a state is to the rare event. Partition the range of $f_I$ into $k+1$ non-empty intervals to obtain a *level function* $f_L\colon S \to \{\, 0,\ldots,k \,\}$ with $f_L(s_1) < f_L(s_2) \Rightarrow f_I(s_1) < f_I(s_2)$. For simplicity, assume $f_I(s_0) = 0$ and $\mathit{step}(s) = \langle t, s'\rangle \Rightarrow f_L(s') \leq f_L(s)+1$ (a step moves up by at most one level). Let $C_i \overset{\text{def}}{=} \{\, s \mid f_L(s) \geq i \,\}$. Then "thresholds $T_i$ of $f_I$ are defined so that each set $C_i$ is associated

**Input:** model $\langle S, s_0, step, F\rangle$, $f_L$, $f_S$, prolongation depth $j$, max. sim. time $T_{max}$

```
t_F := 0, list ξ := {| ⟨s_0, 0, 0, 0⟩ |}            // ⟨state, time, creation level, last-split level⟩
while ξ ≠ ∅ do                                        // run all trials to end
    ⟨s, t, ℓ_create, ℓ_split⟩ := ξ.get-remove()       // data of current trial
    while t < T_max do
        ⟨t', s'⟩ := step(s)                           // simulate to next change in state
        t' := min{ t', T_max − t }, t := t + t'       // advance time, at most to T_max
        if s ∈ F then t_F := t_F + t' / ∏_{i=1}^{ℓ_split} f_S(i)   // accumulate weighted rare time
        ⟨ℓ, ℓ'⟩ := ⟨f_L(s), f_L(s')⟩, s := s'
        if ℓ' < ℓ then                                // trial went down:
            if ℓ' = 0 = ℓ_create then ℓ_split := 0    // reset main trial at level 0
            else if ℓ' = 0 ∨ ℓ' < ℓ_create − j then break   // end retrial if 0 or j down
            else ℓ_split := min(ℓ_split, ℓ' + j)      // else update last-split level
        else if ℓ' > ℓ_split then                     // trial went up far enough:
            ℓ_split := ℓ'                             // update last-split level
            foreach i ∈ {1, ..., f_S(ℓ')−1} do ξ.add(⟨s', t, ℓ', ℓ_split⟩)   // split off retrials
return t_F                    // return accumulated weighted time spent in rare states
```

**Algorithm 1:** RESTART with prolonged retrials of depth $j$ (RESTART-P$_j$)

with $f_I \geq T_i$" [21]. Function $f_S\colon \{1,\dots,k\} \to \mathbb{N} \setminus \{0\}$ defines *splitting factors.* $f_I$, $f_L$, and $f_S$ are specified by experts or derived automatically [6]. Importance splitting with RESTART starts a run (the *main trial*) from $s_0$ that, whenever it moves up from $s$ in current level $l-1$ to $s'$ in level $l$, spawns $f_S(l)-1$ new child runs (*retrials of level* $l$) from $s'$. Retrials end when they move down below their creation level. The trials' weights in probability estimation are appropriately reduced to compensate. RESTART with prolonged retrials of depth $j$, denoted RESTART-P$_j$, is defined as follows in [21] (shortened and adapted to our notation):

> In RESTART-P$_j$, each of the retrials of level $i$ finishes when it leaves set $C_{i-j}$; that is, it continues until it down-crosses the threshold $i-j$. If one of these trials again up-crosses the threshold where it was generated (or any other between $i-j+1$ and $i$), a new set of retrials is not performed. If $j \geq i$, the retrials are cut when they reach the threshold 0. The main trial, which continues after leaving set $C_{i-j}$, potentially leads to new sets of retrials if it up-crosses threshold $T_i$ after having left set $C_{i-j}$. If the main trial reaches the threshold 0, it collects the weight of all the retrials (which has been cut at that threshold) and thus, new sets of retrials of level 1 are performed next time the main trial up-crosses threshold $T_1$.

In addition, [21, Fig. 1] graphically illustrates the behaviour of RESTART-P$_1$. The original RESTART [22] is RESTART-P$_0$. The above textual description clearly conveys the core idea of RESTART-P, but we found it to omit three technical details:

– The condition for when an up-going retrial spawns new retrials is more complex than with RESTART. We became aware of this when comparing the textual description with the graphical depiction in [21, Fig. 1]. In fact, we need to keep track of the last level at which a retrial will split, and decrement that value when it moves more than $j$ levels down. (Independent replication.)
- The definitions in [19,21] do not include 0 in the range of values for $i$ in $C_i$ and $T_i$. Our definitions would associate $T_0$ with states $s$ where $f_I(s) = 0$. Implemented in FIG, this lead to increasing underestimation as the prolongation depth $j$ increased. Only once we interpreted threshold 0 to refer to *level* 0 (i.e. states $s$ where $f_L(s) = 0$) did we obtain consistent estimations across different $j$. The correctness of this interpretation was confirmed by the author of [19,21] in private communication. (Semi-independent replication.)
- When a trial reaches, or spends time in, a state in $F$, we must weight this event's influence on the statistical estimate by a factor of $1/\prod_{i=1}^{f_L(s)} f_S(i)$ in the original Restart. With this weight calculation, FIG produced subtle underestimations on some of the models from [20] when $j > 0$. We finally requested the source code for the original experiments and found that $f_L(s)$ must be replaced by *the level on which the current trial was last split*, i.e. the value must not change when moving down $\leq j$ levels. (Resembles a reproduction.)

We make these details explicit in Algorithm 1, for the case of estimating the long-run average time spent in $F$ (i.e. steady-state simulation). FIG evolved as described above and is thus mostly a replication. modes was extended with prolongations later, using a recursive formulation of the algorithm gleaned from the original code. It thus lacks the complete independence of a replication as per [1].

## 3 Experiments

Table 2 in [21] provides steady-state estimates, numbers of samples, and runtimes obtained using Restart-P$_j$ on a Jackson (i.e. Markov) 2-tandem queueing network for $j \in \{0, \ldots, 4\}$. The same data is given in [19] for $j \in \{0, \ldots, 2\}$ on a similar system with three queues and a seven-node network, in Jackson and non-Jackson (using Erlang and hyperexponential distributions) variants. The original articles and extra material [20] describe the models, and the experimental setup:
- The set $F$ is characterised. E.g. for the 3-tandem network, it contains the states where the third queue has $\geq L = 30$ (Jackson) or 14 packets (non-Jackson).
- All probability distributions and the $f_I$, $f_L$, and $f_S$ functions are characterised.
- $T_{max}$ time values for the steady-state simulations are specified for all models.
- The statistical evaluation aims for a relative error of 0.1 with 95 % confidence (except for the tandem queue, where the error is 0.005); Restart-P runs are performed sequentially until the half-width of the 95 % confidence interval is below 10 % (resp. 0.5 %) of the current estimate. (Note that this guarantees the requested confidence only asymptotically for decreasing width [9].)

In our replication attempt, we had to resolve the following unspecified aspects:
- The queue capacities $C > L$ are not documented, but influence the estimate: for $C$ close to $L$, the steady-state probability is underestimated. We settled for $C = 20 \cdot L$ in FIG's IOSA models (replication); the influence of $C - L$ rapidly diminishes beyond small values. Later, from inspecting the original source

**Table 1.** Experimental results for the examples considered in [19,21]

| model (type) $p$ | $j$ | original [19,21] $\hat{p}$ | $n$ | time | adapted orig. code $\hat{p}$ | $n$ | time | modes $\hat{p}$ | $n$ | time | FIG $\hat{p}$ | $n$ | time |
|---|---|---|---|---|---|---|---|---|---|---|---|---|---|
| 2-tandem | 0 | 4.85E-15 | 3909 | 2906 | 4.84E-15 | 2731 | 1930 | 4.88E-15 | 2542 | 988 | 4.85E-15 | 2537 | 4202 |
| (Jackson) | 1 | 4.86E-15 | 3032 | 2107 | 4.93E-15 | 1905 | 1654 | 4.87E-15 | 1859 | 939 | 4.82E-15 | 1969 | 4000 |
| 4.86E-15 | 2 | 4.86E-15 | 2660 | 2091 | 4.80E-15 | 1831 | 1959 | 4.85E-15 | 1845 | 1175 | 4.86E-15 | 1700 | 4379 |
| | 3 | 4.87E-15 | 2476 | 2287 | 4.86E-15 | 1691 | 2319 | 4.83E-15 | 1626 | 1322 | 4.84E-15 | 1656 | 5448 |
| | 4 | 4.85E-15 | 2458 | 3188 | 4.88E-15 | 1562 | 2638 | 4.85E-15 | 1610 | 1626 | 4.86E-15 | 1580 | 6402 |
| 3-tandem | 0 | 4.66E-15 | 120 | 54 | 4.90E-15 | 89 | 28 | 4.24E-15 | 116 | 9 | 4.58E-15 | 122 | 43 |
| (Jackson) | 1 | 4.61E-15 | 88 | 35 | 4.84E-15 | 44 | 20 | 4.90E-15 | 97 | 10 | 5.63E-15 | 80 | 36 |
| 4.86E-15 | 2 | 4.66E-15 | 78 | 38 | 4.84E-15 | 49 | 19 | 4.83E-15 | 79 | 11 | 5.23E-15 | 65 | 39 |
| 3-tandem | 0 | 7.08E-15 | 95 | 137 | 8.38E-15 | 728 | 180 | 8.87E-15 | 1002 | 256 | 8.28E-15 | 1293 | 715 |
| (non-J.) | 1 | 7.03E-15 | 65 | 90 | 8.50E-15 | 661 | 181 | 8.10E-15 | 650 | 182 | 8.65E-15 | 618 | 436 |
| | 2 | 7.03E-15 | 55 | 90 | 8.34E-15 | 388 | 191 | 8.53E-15 | 386 | 157 | 9.59E-15 | 386 | 402 |
| 7-nodes | 0 | 2.53E-15 | 42 | 16 | 2.33E-15 | 44 | 18 | 2.59E-15 | 36 | 10 | 2.34E-15 | 52 | 277 |
| (Jackson) | 1 | 2.46E-15 | 28 | 12 | 2.50E-15 | 34 | 14 | 2.34E-15 | 26 | 11 | 2.47E-15 | 32 | 248 |
| 2.54E-15 | 2 | 2.46E-15 | 27 | 12 | 2.41E-15 | 20 | 13 | 2.63E-15 | 25 | 15 | 2.42E-15 | 32 | 332 |
| 7-nodes | 0 | 7.57E-15 | 54 | 56 | 7.96E-15 | 149 | 52 | 8.98E-15 | 135 | 88 | 8.55E-15 | 202 | 1305 |
| (non-J.) | 1 | 7.40E-15 | 44 | 52 | 7.37E-15 | 92 | 45 | 7.46E-15 | 103 | 84 | 8.03E-15 | 142 | 1323 |
| | 2 | 7.64E-15 | 30 | 32 | 7.29E-15 | 79 | 52 | 8.31E-15 | 91 | 119 | 7.45E-15 | 126 | 1495 |

code, we found that the queues are practically unbounded (implemented as 32-bit integer counters), which we reproduce in the MODEST models for modes.

- FIG by default uses the *batch means* technique for steady-state simulation, where a single run is partitioned into equal-duration batches, each of which provides one sample value. In communication with the original author, we found that each of their samples results from an independent run. We adapted FIG to do the same. It is the default in modes. (Semi-independent replication.)
- We also found in this communication that the original runs perform no splitting for the first 40 clients served; this part of the run is ignored as an initial transient phase. We confirmed this in the source code. We measured the average time to serve 40 clients for each model and use the result as transient phase *duration* with FIG and modes since neither tool supports a transient phase based on clients served. (Semi-independent replication.)

The original experiments were realised in a single file of C code that represents both the algorithm and the models, specialised to queueing models with transition probabilities and service rates specified in constant arrays. In fact, the code we received implemented the 2-tandem queueing network only. We extended this code with a compile-time choice among the models described in [20], and fixed few small bugs. We thus have four sets of results to compare, shown in Table 1: the original numbers given in [19,21], plus those from our new executions of the adapted code, modes, and FIG. In the table, time is in seconds, $\hat{p}$ is the estimate, $p$ is the true steady-state probability where it can be derived, and $n$ is the number of samples needed by the statistical evaluation. The adapted code and FIG ran on an Intel Xeon E5-2630 v3 (2.4-3.2 GHz), and modes ran on a Core i7-4790

(3.6-4.0 GHz, 4 physical/8 logical cores) system. The adapted code and FIG are single-threaded whereas modes used 7 simulation threads. The adapted code is tailor-mode for these models, while FIG has to encode them in the more general IOSA framework, making it slower; modes in turn profits from a special-case implementation for CTMC to speed up the Markovian cases. Comparing runtimes *between tools* is thus of limited use. The estimates are the centers of confidence intervals returned by the tools with confidence and relative width as described above. Each $\langle$estimate, $n$, time$\rangle$ triple was selected from 5 tool executions by picking the one with the median runtime. We underline the best runtimes among values for $j$. However, the wide confidence intervals (except for 2-tandem), few executions, and in principle unsound stopping criterion that we reproduce from the original experiments mean that results, including best values of $j$, vary a lot for different random seeds. The original experimental setup is thus insufficient for drawing conclusions about the precise tradeoffs between specific values of $j$, but may at most expose an overall trend.

Nevertheless, our estimates are mostly within the margin of error around the original or true results. We confirm the main experimental conclusion of [19,21]: as $j$ increases, $n$ decreases, but from some point—mostly $j > 1$ or 2—runtime increases, due to the overhead of more retrials surviving longer. For the non-Jackson triple tandem network, none of our results matches the numbers of [19]. Since *the original code*, albeit adapted, agrees with FIG and modes rather than with the original results, we suspect an error in [19] or [20] w.r.t. this one model.

## 4 Conclusion

We demonstrated the extension of the FIG and modes rare event simulation tools to support prolonged retrials in rare event simulation using Restart importance splitting. These implementations and experiments were the outcome of an exercise in independently *replicating* experimental research originally performed in mathematics, from a computer science perspective. We confirm the key findings of the earlier work. At the same time, we document several issues—small but critical technical details of the algorithm and experimental setup—where the publicly available information was insufficient for a completely independent replication. We in particular noticed that replicating randomised/statistical algorithms poses a particular challenge since small errors may result in subtle mis-estimations that are often drowned in the overall statistical error. In the end, however, all issues could be resolved due to the exceptional support, responsiveness, and openness of the original author, José Villén-Altamirano, whom we thank earnestly. However, such support cannot be expected for experimental work in general, in particular where temporary staff like Ph.D. students—who eventually graduate and move to new institutions or industry—perform the bulk of the experiments. This paper thus also highlights the need for computer science and the formal verification community to continue their push for artifact evaluation and archived, publicly available *reproduction* packages. A reproduction package for our experiments is archived at DOI 10.6084/m9.figshare.12269462.
